# Conduits and Strategies for Arterial Revascularization in CABG

**DOI:** 10.31083/j.rcm2407188

**Published:** 2023-06-30

**Authors:** Talal Alzghari, Arnaldo Dimagli, Kevin R. An, Gianmarco Cancelli, Lamia Harik, Roberto Perezgorvas-Olaria, Giovanni Jr. Soletti, Mario Gaudino

**Affiliations:** ^1^Department of Cardiothoracic Surgery, Weill Cornell Medicine, New York, NY 10065, USA

**Keywords:** CABG, coronary artery bypass grafting, internal thoracic artery, radial artery, multiple arterial grafting

## Abstract

Ischemic heart disease is the leading cause of death in the United States. Depending on the severity of the coronary artery disease, treatment options include percutaneous coronary intervention (PCI) and coronary artery bypass grafting (CABG). Although CABG has been performed since the 1970s, there is still debate onwhich conduit to use after the left internal thoracic artery. Currently, national registries report the left internal thoracic artery and the saphenous vein as the most commonly used conduits in CABG, while other arterial grafts, such as the radial artery, represent a minority, even though the current evidence suggests potential benefits of these arterial conduits. In this review, we aimed to describe the different types of arterial conduits used for CABG and summarize the evidence behind their use.

## 1. Introduction

Ischemic heart disease is the leading cause of death in the United States [1]. Treatment options encompasses both percutaneous coronary intervention (PCI) and coronary artery bypass grafting (CABG). The choice between the two depends on the number of involved coronary arteries, the location of involvement and the severity of the stenotic lesions, together with patient comorbidities, such as diabetes [2, 3]. PCI is currently being used for less severe disease [4], while in patients with more complex, multi-vessel coronary artery disease (CAD) evidence has shown better cardiac outcomes and possibly lower long-term mortality with CABG [3, 4, 5]. However, there persists a debate on the best conduits to use to complement the left internal thoracic artery (LITA).

Historically, the first conduit used to bypass coronary lesions was the LITA by Vineberg when he implanted the LITA into the left ventricle directly [6]. After that, David Sabiston from John’s Hopkins described the first direct hand-sewn coronary anastomosis, when he anastomosed a saphenous vein graft (SVG) to the right coronary artery (RCA) [7]. Few years later, George Green performed the first LITA-left anterior descending artery (LAD) anastomosis in New York City [8]. The first off pump LITA to LAD anastomosis was described by Kolesov in 1967 in Russia [9]. Around that time, Rene Favaloro from Cleveland Clinic reported a landmark study presenting more than 300 cases of CABG using SVG which showed excellent outcomes confirmed by angiogram, opening the modern era of CABG surgery [10]. Currently, national registries report the left internal thoracic artery and the saphenous vein as the most commonly used conduits in CABG with more than 90% of CABG performed in the US and Canada using this graft configuration as reported by the Society of Thoracic Surgeons (STS) database [11, 12], while other arterial grafts represent a minority [13, 14]. In this review, we aimed to describe the available arterial conduits used for CABG and summarize the evidence behind their use, with particular attention to multiple and total arterial grafting.

## 2. Internal Thoracic Artery (ITA)

The use of LITA was described in the first era of myocardial revascularization, starting with the Vineberg procedure [6] and Green’s [8] description of LITA-LAD bypass, however, it has been the primary arterial conduit for surgical myocardial revascularization since Loop *et al*. [15] published a study demonstrating a long-term benefit in terms of mortality, myocardial infarction (MI) and cardiac reintervention in patients receiving a single ITA to the LAD compared with patients receiving only venous grafts. Further studies have later corroborated this evidence, reporting also better long-term graft patency [16, 17], reduced risk of recurrent angina, and improved survival with LITA [18]. Decades of data mostly from observational studies have supported the use of LITA over SVG to graft the LAD unless there are specific contraindications to LITA use [15, 19, 20]. Now, the use of LITA for revascularization of the LAD is the gold standard for CABG [3, 21].

Morphologically, the ITA is resistant to atherosclerosis due to its fewer endothelial fenestrations, lower intercellular junction permeability, and greater anti-thrombotic molecules such as heparin sulfate and tissue plasminogen activator, it also produces higher levels of nitric oxide compared to other arterial and venous grafts [18]. These characteristics might be the biological reason for the higher patency rates of the ITA compared to the SVG.

In cases of severe and multiple CAD the use of more than one arterial graft has been encouraged. Right internal thoracic artery (RITA) has often been used as a second arterial conduit, and its use is a Class 2a recommendation in the 2021 ACC/AHA/SCAI guidelines on coronary artery revascularization [3]. However, there are several factors that should be considered when choosing grafts for non-LAD targets, in particular, life expectancy, severity of the target stenosis, graft quality, sternal wound complication risk factors (e.g., diabetes, chronic obstructive pulmonary disease (COPD)), and surgical expertise.

The RITA is histologically and biologically equivalent to LITA [22]. Tatoulis *et al*. [17] reported 15-year patency rate of RITA as >90%. However, when RITA patency was compared to SVG at 1 year it showed no statistical difference (97.9% vs 96.9%; SVG vs RITA, *p* = 0.36) [23], whereas RITA showed superior patency at 4 years (95% vs 90%; RITA vs SVG, *p* = 0.001) [24]. Gaudino *et al*. [25] in a recent meta-analysis of 14 randomized controlled trials comparing the angiographic patency rates of the bypass conduits showed that the pooled patency of 399 RITA grafts is 90.9% (95% confidence interval [CI], 72.1%–97.5%) at pooled angiographic follow-up period of 6.9 years (Table 1, Ref. [25]), although the rate of graft occlusion difference compared to SVG was not statistically significant (Incidence rate ratio [IRR], 1.02; 95% CI, 0.63–1.65).

**Table 1.** **Pooled angiographic patency rates of different arterial conduits in a meta-analysis of 14 studies including 3396 patients**.

Conduit type	Number of studies	Number of grafts assessed	Pooled patency rate (95% CI)	Angiographic follow-up in years
Right internal thoracic artery	5	399	90.9 (72.1–97.5)	6.9
Radial artery	11	1178	93.2 (87.4–96.4)	5.5
Right gastroepiploic artery	2	136	61.2 (52.2–69.4)	2.8

(Reproduced from Gaudino M., *et al*. [25] Angiographic Patency of Coronary Artery Bypass Conduits: A Network Meta-Analysis of Randomized Trials. J Am Heart Assoc. 2021).

Multiple factors other than the histological and biological nature of the conduit itself play major roles in conduit patency like target coronary vessel, degree of target coronary vessel stenosis, surgical techniques, and competitive flow. For example, Tatoulis *et al*. [17] reported a patency of 95% for the RITA-LAD while lower patency rates were described for RITA-circumflex artery (91%) and RITA-RCA patency (84%) at 10 years.

RITA can be used as an *in situ* graft, a free graft as aorto-coronary graft, or as a composite graft with LITA. In a study including 991 angiograms from patients who received either *in situ* RITA or free RITA (FRITA), Tatoulis *et al*. [26] reported no difference in 10-year patency rates of *in situ* RITA compared to FRITA (89% vs 91%, *p* = 0.44). Similarly, Fukui *et al*. [27] reported no significant difference in angiographic patency rates between *in situ* and FRITA at 1 year (*in situ* 95.3% vs FRITA 89.8%, *p* = 0.17). In a randomized prospective study of 304 patients, Glineur *et al*. [28] reported similar angiographic graft patency when they compared *in situ* bilateral internal thoracic arteries (BITA) to RITA used as a composite Y-graft at 6 months follow-up (96% vs 97%, *p* = 0.69), and in a later analysis at 3-year follow-up (93% vs 94.5%, *p *= 0.81) [29].

Different studies have shown that the degree of target vessel stenosis is a major factor affecting graft patency. Tatoulis *et al*. [26] reported that RITA patency at a mean follow-up of 100 months was 73.9% when target vessel stenosis was <60% and 91.6% when the stenosis was >60%. Sabik *et al*. [30] reported that ITA patency was inversely associated with the degree of native coronary artery stenosis; at 15-year follow-up the patency of ITA was 93% when grafting LAD with 50% stenosis and 98% for LAD lesions with >90% stenosis. When ITA was used to graft non-LAD territories, patency was 76% if target stenosis was 50% and 93% if stenosis was >90%.

Several observational studies reported clinical benefits with the use of BITA as CABG conduits [31, 32], Puskas *et al*. [33] in a propensity adjusted analysis of 3527 CABG of which 812 BITA and 2715 single ITA (SITA) in diabetic and nondiabetic patients showed significantly lower rates of major adverse cardiac events (MACE) in nondiabetic patients with BITA but no difference in diabetic patients and a 35% reduction in long-term death at 15 years with BITA vs SITA. Buttar *et al*. [34] in a meta-analysis including 89,399 patients, with 20,949 patients undergoing BITA and 68,450 patients receiving LITA grafting found that patients in whom BITA were used, compared with those receiving only LITA, had statistically significant better long-term survival (hazard ratio [HR], 0.78; 95% CI, 0.72–0.84, *p* < 0.001) and lower in-hospital mortality (1.2% vs 2.1%, *p* = 0.04), cerebrovascular accidents (1.3% vs 2.9%, *p* = 0.0003), and need for revascularization (4.8% vs 10%, *p* = 0.005), while the incidence of deep sternal wound infection (DSWI) was higher (1.8% vs 1.4%, *p* = 0.0008).

The Arterial Revascularization Trial (ART) is the only large randomized BITA trial including 28 centers, 7 countries and 3102 patients randomly assigned to the use of BITA (1548 patients) vs SITA (1554 patients); in the 10-year intention-to-treat analysis there was no difference in mortality or event free survival comparing BITA to SITA (HR, 0.96; 95% CI, 0.82–1.12 and HR, 0.90; 95% CI, 0.79–1.03, respectively) [35]. There are, however, important issues with the ART trial including a high degree of crossover from the BITA arm (16.4%) which was 4-fold higher than from the SITA arm (3.9%). The rate of crossover varied between 0–100% among the 131 operating surgeons [36]; this high rate of crossover did not only dilute the potential effect of BITA, but it may also indicate that some surgeons lacked confidence with BITA conduits, as it has been shown that BITA grafts outcomes are highly dependent on the operating surgeon’s volume and experience [37, 38]. Another important issue was the high rate of RA use in the SITA group (21.8%). In summary, only 1330/1554 patients of LITA group received a single arterial graft and only 1294/1531 patients of BITA group actually received BITA. In an observational as-treated analysis of patients who received multiple arterial grafts (MAG) had significantly lower risks of mortality and major adverse cardiac events compared to patients who received single arterial graft (SAG) (adjusted HR, 0.81; 95% CI, 0.68–0.95 and adjusted HR, 0.80; 95% CI, 0.69–0.93).

One of the major concerns with the use of BITA grafts is substantially increased risk of sternal wound complications. Dai *et al*. [31] in a meta-analysis of 172,880 patients found that BITA use compared to SITA was associated with higher risks of sternal wound complications (SITA:1.6% vs BITA: 2.05%; relative risk [RR], 1.61; 95% CI, 1.4–1.81, *p* = 0.05) and higher risks in diabetic patients (SITA: 1.66% vs BITA: 2.64%; RR, 0.65; 95% CI, 0.52–0.81, *p* = 0.05). It is thought that careful patient selection and surgical harvest technique of the ITA (i.e., skeletonization) significantly reduce the risk of deep sternal wound infection even in patients with diabetes [31, 32] as we discuss below in the harvesting techniques section. Table 2 (Ref. [3]) summarizes 2021 ACC/AHA/SCAI guideline recommendations for arterial conduits.

**Table 2.** **Arterial conduits best practices in CABG from (2021 ACC/AHA/SCAI Guideline for Coronary Artery Revascularization) [3]**.

Conduit type	Best practices
Right internal thoracic artery	- Skeletonized harvesting of the ITA reduces the risks of sternal wound infections
Radial artery	- Objective assessment of the palmar arch and ulnar compensation before harvest
	- Choose target vessels with sub-occlusive stenosis
	- Avoid the use of the RA after trans-radial catheterization
	- Avoid the use of RA in patients with CKD and a high likelihood of rapid progression to hemodialysis
	- 1 year of oral calcium channel blockers postoperatively
	- Avoid bilateral percutaneous or surgical RA interventions in CAD patients to preserve it for the future if needed
Right gastroepiploic artery	- Use the skeletonized RGEA to graft RCA target vessels with sub-occlusive stenosis if the operator is experienced

CABG, Coronary Artery Bypass Graft; ITA, Internal Thoracic Artery; RA, Radial Artery; RGEA, Right Gastroepiploic Artery; RCA, Right Coronary Artery; CKD, Chronic Kidney Disease; CAD, Coronary Artery Disease.

## 3. Harvest Technique

A variety of surgical techniques can be used to harvest the ITAs, including open sternotomy, small anterolateral chest incision and ITA takedown under direct vision, thoracoscopic or robotic harvest. Classical median sternotomy is generally used if total arterial revascularization is planned, as it allows for excellent exposure of the bilateral ITA, and the target vessels [39].

Either ITA can be harvested as a pedicle with the endothoracic fascia and paired veins or as a skeletonized graft. Although skeletonization consumes more time and is more technically demanding, it is thought that it is associated with lower risks of sternal complications especially in patients with bilateral ITA grafts. Compared to pedicled grafts, skeletonized grafts were shown to be significantly better in terms of length, caliber, and flow velocity [40].

In a meta-analysis of 129,871 patients (124,233 LITA, 5638 BITA), Zhou *et al*. [41] found that although patients with BITA had significantly higher rate of DSWI compared to LITA (3.26% for BITA vs 1.70% for LITA, *p <* 0.001), there was no significant difference between the 2 groups when the grafts were skeletonized (2.46% for LITA vs 2.48% for BITA, *p *= 0.84). However, Gaudino *et al*. [42] in a post-hoc analysis of ART comparing skeletonized and pedicled ITA including 2161 patients reported no significant difference in all-cause mortality between 2 groups (HR, 1.12; 95% CI, 0.92–1.36, *p *= 0.27), but an increased risk of MACE in patients with skeletonized graft compared to the pedicled graft (HR, 1.25; 95% CI, 1.06–1.47, *p* = 0.01).

Even with the presence of this contradicting data the use of skeletonized ITA in BITA grafting is recommended as best practice in the 2021 ACC/AHA/SCAI guidelines of coronary artery revascularization [3]. Table 3 (Ref. [3]) presents 2021 ACC/AHA/SCAI best practice recommendations to decrease the risk of sternal wound infections.

**Table 3.** **Best practices to reduce risk of sternal wound infections in patients undergoing CABG from (2021 ACC/AHA/SCAI Guideline for Coronary Artery Revascularization) [3]**.

Nasal swab for Staphylococcus aureus
Nasal carriers of S. aureus, use mupirocin 2% ointment
In patients with unknown nasal culture or PCR, use preoperative intranasal mupirocin 2%
For long procedures (>2 half-lives of the antibiotic) or in cases of excessive blood loss during CABG, readminister the prophylactic antibiotics
Obtain HbA1c perioperatively
Treat any infection before nonemergent CABG
Recommend smoking cessation preoperatively
Apply topical vancomycin to sternal edges after opening and before closing in all cardiac surgeries
Harvest skeletonized graft in BITA grafting
Stop antibiotics in 2 days

BITA, bilateral internal thoracic artery; CABG, coronary artery bypass graft; HbA1c, glycated hemoglobin A1c; and PCR, polymerase chain reaction.

## 4. Radial Artery (RA) 

The use of RA was first introduced to coronary bypass surgery by Carpentier *et al*. [43] in the 1970s, but due to the high rates of early graft failure caused by diffuse narrowing and intimal hyperplasia it was soon abandoned [44, 45, 46]. Almost 2 decades later the idea of RA use was revived by Acar *et al*. [47], and thereafter multiple studies have shown that the change in the harvesting technique and the use of antispasmodic medications improved the short- and mid-term patency [47, 48, 49], especially when the RA is anastomosed to a target vessel with high-grade stenosis [50].

Observational studies have shown that the RA has a patency rate of >90% at 10 years [51] and >85% at 20 years when target vessel stenosis is ≥90% [52]. Gaudino *et al*. [25] in a recent meta-analysis, of 14 randomized controlled trials comparing the angiographic patency rates of the bypass conduits showed that the pooled patency of 1178 RA grafts at a follow-up of 5.5 years was 93.2% (95% CI, 87.4–96.4) Table 1, and the RA was associated with significantly lower graft occlusion rate compared to SVG (IRR, 0.54; 95% CI, 0.35–0.82).

When comparing RA to SV clinically, Gaudino *et al*. [53] reported in a meta-analysis of 14 adjusted observational studies including 20,931 patients that at a mean follow-up of 6.6 years the mortality was significantly lower in the RA group when compared to the SV group (24.5% in RA vs 34.2% in SV group; IRR, 0.74; 95% CI, 0.63–0.87, *p* < 0.001). In a patient-level pooled analysis comparing the use of the RA vs SV as a second conduit for CABG and including a total of 1036 patient randomized to RA (n = 534) or SV (n = 502), the Radial Artery Database International Alliance (RADIAL) consortium [54] investigators found that the use of the RA when compared to the SV at 10 years follow-up was associated with significantly lower incidence of primary composite outcome of death, MI or repeat revascularization (HR, 0.73; 95% CI, 0.61–0.88, *p* < 0.001) and in the composite outcome of death and MI (HR, 0.77; 95% CI, 0.63–0.94, *p* = 0.01).

The Radial Artery Patency and Clinical Outcomes (RAPCO) program included two separate trials in which patients, based on their age and diabetes status, were randomized to RA vs FRITA or RA vs SV. The goal for the RA vs FRITA arm was to compare between both conduits when they were used as a direct aorto-coronary graft to bypass the second most important lesion after LITA-LAD in patients <70 years or diabetic patients <60 years. At 10 years, RAPCO investigators reported significantly higher patency for the RA compared to the FRITA (89% for RA vs 80% for FRITA; HR, 0.45; 95% CI, 0.23–0.88), and better patency for RA when compared to SV (85% for RA vs 71% for SV; HR, 0.4; 95% CI, 0.15–1.00). The RAPCO trial was limited in that only 80% of patients in the RITA vs RA and 65% in the SV vs RA comparisons had imaging follow-up, and the follow-up imaging timing was randomly assigned to the patients [55]. In November 2022, Hare *et al*. [56] presented the 15-year follow-up of both arms of RAPCO at the American Heart Association Scientific Session, which showed significantly lower incidence of the composite outcome of all-cause mortality, MI, or repeat revascularization in the RA vs FRITA (39.4% vs 48.5%, *p* = 0.04) and in the RA vs SV (60.2% vs 73.2%, *p* = 0.04).

One meta-analysis compared the angiographic studies of the RA and RITA, reported that RITA was associated with nonsignificant 27% absolute risk reduction for late functional graft occlusion [14].

In the 2021 ACC/AHA/SCAI guidelines of coronary artery revascularization; the use of RA is a Class I recommendation to graft the second most important, significantly stenosed, non-LAD target vessel [3]. Table 2 summarizes 2021 ACC/AHA/SCAI best practices recommendations for arterial conduits.

## 5. Harvest Technique

The RA is traditionally harvested from the non-dominant arm, although there is no data to support this practice. In small series, bilateral RA harvest has been shown to be safe with minimal to no clinical sequelae or hand function compromise [57, 58, 59].

The adequacy of the ulnar artery collateral flow is generally assessed before harvesting, but there is no standard method for assessment. The modified Allen test is a simple bedside examination that helps in assessing ulnar artery and collateral flow adequacy but its’ clinical reliability has frequently been questioned [60]. The use of supplementary pulse oximeter in conjunction with the Allen test has often been proposed to improve accuracy [61] and the supplementary use of ultrasonography has also been described [62]. The preoperative use of echo-doppler ultrasound is useful especially with providing morphometric information on the RA.

Traditionally the RA is harvested as a pedicle with the vena comitans and surrounding connective tissues [47]. Skeletonization of the RA [63], involves meticulous dissection of the vessel free from supporting tissues and veins. This allows to have a longer conduit which can be used for sequential anastomoses or composite grafts. Moreover, it increases the luminal diameter and minimizes the risk of graft kinking. Also, due to the absence of periarterial tissues it allows a better assessment of the graft, such as presence of hematomas, and of the anastomosis and facilitates the topical application of vasodilative drugs [63].

On the other hand, the RA tends to spasm easily, therefore during the graft manipulation, preservation of the endothelium and reduction of mechanical stimuli are essential to reduce the spasm. For these reasons, a pedicled RA is usually used. In a study comparing the RA harvesting (skeletonized vs pedicled) using scissors and clips or the ultrasonic scalpel, electron microscopy images revealed endothelial damages in all the grafts, regardless of the technique and instruments used; however, severe endothelial injuries were more likely present when the RA was skeletonized and harvested with the ultrasonic scalpel. Conversely, endothelial damage was the lowest in pedicled RAs harvested with the ultrasonic scalpel [64]. Skeletonization is also more technically demanding and requires longer harvesting time. There is very limited evidence comparing the patency rates of RA harvested with the two techniques [65, 66].

The RA can be harvested using open or endoscopic technique. There is no clear evidence of differences in patency or clinical outcomes between harvesting techniques, but endoscopic harvesting is cosmetically superior and associated with less local wound complications (e.g., wound infection, hematoma formation and paresthesia) compared to the open technique [67].

Technical benefits of the RA compared to other arterial conduits include the possibility to harvest it simultaneously with the LITA therefore reducing the overall operative times. Also, the RA is longer, more versatile, and easier to handle due to its diameter and wall thickness. It can be used as an aorto-coronary graft, “I” or “Y” composite graft, or as an extension for a short LITA or RITA graft, and due to its length it can be used potentially for any target lesion (anterior, lateral, or posterior). In addition, the RA is useful in diabetic patients with concerns of sternal wound complications if BITA was used, or in patients with ambulation concerns especially after SV harvest, or in patients with venous conduit shortage.

Contraindications to RA use are diameter <2 mm or diffuse calcification; absence of flow reversal in the RA during compression, <20% increase in ulnar artery (UA) peak systolic flow during RA compression, and >40% decrease in digital pressure during RA compression at digital plethysmography [68]. It is also contraindicated in patients with distal arteriovenous fistula hemodialysis access, and in patients with Raynaud’s disease or vasculitis [59, 69].

## 6. Right Gastroepiploic Artery (RGEA)

In the early 1980’s, Pym *et al*. [70] reported the first systematic use of RGEA graft, since then RGEA has been used as an alternative arterial conduit for CABG. Histologically, the RGEA when compared to ITA has more smooth cells in its media, resulting in more spastic response when handled surgically [71], it also responds more powerfully to vasoactive medications compared to ITA [72]. However, its blood flow increases postprandially [73].

Suma *et al*. [74] reported their 20-year experience with GEA as a conduit for CABG in 1352 patients, which showed patency rate of 97.1% at 1 month, 92.3% at 1 year, 85.5% at 5 years, and 66.5% at 10 years. In 172 skeletonized RGEA grafts with 233 distal anastomoses, the patency rate at immediate, 1, and 4 years was 97.6%, 92.9%, and 86.4%, respectively. Skeletonization of RGEA with anastomosis to target vessel with >90% as used by Suzuki *et al*. [75] resulted in patency rates of 97.8% in early post operative period, 94.7% at 5 years, and 90.2% at 8 years postoperatively. Gaudino *et al*. [25] in a recent meta-analysis of 14 randomized controlled trial (RCT)’s examining angiographic patency of arterial conduits, including 136 GEA conduits, over pooled follow-up period of 2.8 years, reported pooled patency of 61.2% (95% CI, 52.2–69.4) (Table 1), and GEA occlusion rate when compared to SVG it was not statistically significant (IRR, 0.98; 95% CI, 0.57–1.68).

## 7. Harvest Technique

RGEA is usually harvested by extending the median sternotomy incision caudally for about 5–6 cm or through a total separate upper abdominal incision. After opening the peritoneal cavity, the artery is identified, traced and dissected under direct visualization, the in-situ pedicle is then passed cranially through the diaphragm to be anastomosed to the target vessel. The RGEA can also be harvested as a skeletonized graft by opening the greater omentum and dividing the omental and gastric branches, skeletonization can be performed using electrocautery, hemoclips and scissors; the use of the ultrasonic scalpel has been proposed to improve efficiency and reduce harvesting time [76]. Skeletonization of the RGEA graft allow longer conduits and facilitates visual inspection and sequential anastomosis [77] Morimoto *et al*. [78] described harvesting the RGEA laparoscopically. Table 4 (Ref. [68, 79, 80, 81]) summarizes recommendations and limitations of RA and RGEA use.

**Table 4.** **Proposed recommendations and limitations of RA and RGEA use**.

Conduit type	Recommendations	Limitations
Radial artery	- It is recommended to use the RA as a second conduit to bypass the second most important, significantly stenosed, non-LAD target to improve cardiac outcomes	- It is not recommended to use RA with <2 mm diameter or diffuse intimal hyperplasia, absence of flow reversal during manual compression, ulnar artery peak systolic flow increase <20% during RA compression, digital pressure decrease >40% during RA compression [68]
	- RA use should be limited to target vessel stenosis >70% and ideally >90% [79]	- It is discouraged to use recently cannulated RA due to potential endothelial injury [80]
Right gastroepiploic artery	- RGEA could be used to graft the distal RCA or PDA, and it can be helpful in cases of redo-CABG [81]	- Need for a separate incision or extension of the sternotomy incision
		- Blood flow changes in response to meals

RA, Radial Artery; RGEA, Right Gastroepiploic Artery; LAD, Left Anterior Descending; RCA, Right Coronary Artery; PDA, Posterior Descending Artery; CABG, Coronary Artery Bypass Graft.

## 8. Multiple and Total Arterial Grafting

In patients with multi-vessel CAD, the use of 2 or more arterial grafts with possible addition of SVG is referred to MAG or the use of 3 or more arterial grafts with no SVG grafts is referred to as total arterial grafting (TAG). The available evidence has been conflicting about the benefit of a 3rd arterial conduit [82, 83, 84, 85]. Benedetto *et al*. [82] reported no significant long-term survival benefit of BITA + RA vs BITA + SV after a mean follow-up of 10.6 years (97.4%, 90.3% and 81.7% at 5, 10, and 15 years, respectively vs 97.0%, 94.1%, and 82.1%, *p* = 0.54; HR, 1.16; 95% CI, 0.71–1.9), Di Mauro *et al*. [83] reported that BITA + SV was associated with significantly higher 8 years freedom from cardiac deaths when compared to BITA + arterial conduit (RA/RGEA) (98.6% ± 0.5% with SV vs 95.3% ± 1.3% with arterial conduit, *p* = 0.009), whereas Rocha *et al*. [86] reported in a propensity score matching analysis, that TAG compared to non-TAG was associated with improved long-term (8 years) freedom from major adverse cardiac and cerebrovascular events (HR, 0.78; 95% CI, 0.68–0.89), death (HR, 0.80; 95% CI, 0.66–0.97), and myocardial infarction (HR, 0.69; 95% CI, 0.51–0.92). Royse *et al*. [87] in a large cohort including 51,113 CABG cases, reported that the use of any SVG was independently associated with higher mortality rate at average of 12.5 years (matched HR, 1.22; 95% CI, 1.15–1.30, *p* < 0.001 and unmatched HR, 1.24; 95% CI, 1.18–1.30, *p* < 0.001).

In a meta-analysis of propensity matched series, comparing 2 arterial grafts (AG) vs 3 AG including 8 studies and 10,287 matched patients (2 AG: 5346; 3 AG: 4941) Gaudino *et al*. [88] reported that 3 AG were associated with a statistically significantly reduction in the hazard of late death (HR, 0.80; 95% CI, 0.75–0.87, *p* < 0.001), irrespective of sex and diabetes mellitus status. In another meta-analysis including 130,305 patients, Yanagawa *et al*. [89] found that TAG was associated with significantly lower mortality rate when compared to single AG (incidence rate ratio [IRR], 0.85; 95% CI, 0.81–0.89, *p* < 0.0001) or even MAG (IRR, 0.85; 95% CI, 0.73–0.99, *p* = 0.04). In a recent meta-analysis of 14 observational studies including 22,746 patients (TAG: 8941 and non-TAG: 13,805), Rayol *et al*. [90] reported a lower risk of 10 years mortality in TAG vs non-TAG (HR 0.67; 95% CI, 0.58–0.77, *p* < 0.001).

There is limited evidence from RCTs to compare the effects of MAG vs TAG. Muneretto *et al*. [91] randomized 200 patients >70 years old to receive TAG or conventional CABG that includes SVG. At a mean follow-up of 12 ± 4 months, mortality was not different in both groups (5% vs 4%, *p* = 0.99), but a significantly lower recurrent angina (2% vs 13%, *p* < 0.001), or new percutaneous revascularization (0% vs 8%, *p* = 0.012) were found in TAG patients. At multivariable analysis, SVG use was found to be an independent factor for graft occlusion and angina recurrence (odds ratio (OR), 1.16; 95% CI, 1.08–1.23), additionally, SVG use significantly affected late cardiac related events (HR, 2.29; 95% CI, 1.49–3.08, *p* = 0.041).

A post-hoc analysis of the ART trial found that there was an increasing benefit with the use of more arterial grafts, in particular, there was a significant reduction of 10-year mortality (HR, 0.68; 95% CI, 0.47–0.97, *p* = 0.03) and the composite outcome of death/MI/stroke and repeat (HR, 0.71; 95% CI, 0.53–0.94, *p* = 0.02). However, what limits this comparison is that it is observational and nonrandomized in nature [92].

## 9. Graft Configurations 

Multiple arterial grafts configurations have been evolved in the last few decades to achieve total arterial grafting. In-situ grafts and free grafts have been used. In the early 90’s, Tector *et al*. [93] described a technique of total arterial revascularization with “T-grafts” constructed from LITA and free RITA where anterior and anterolateral areas of the heart are bypassed with the LITA and the inferolateral, inferior and posterior areas are bypassed with the RITA. Since then, multiple different graft configurations have been evolved.

**Fig. 1.**
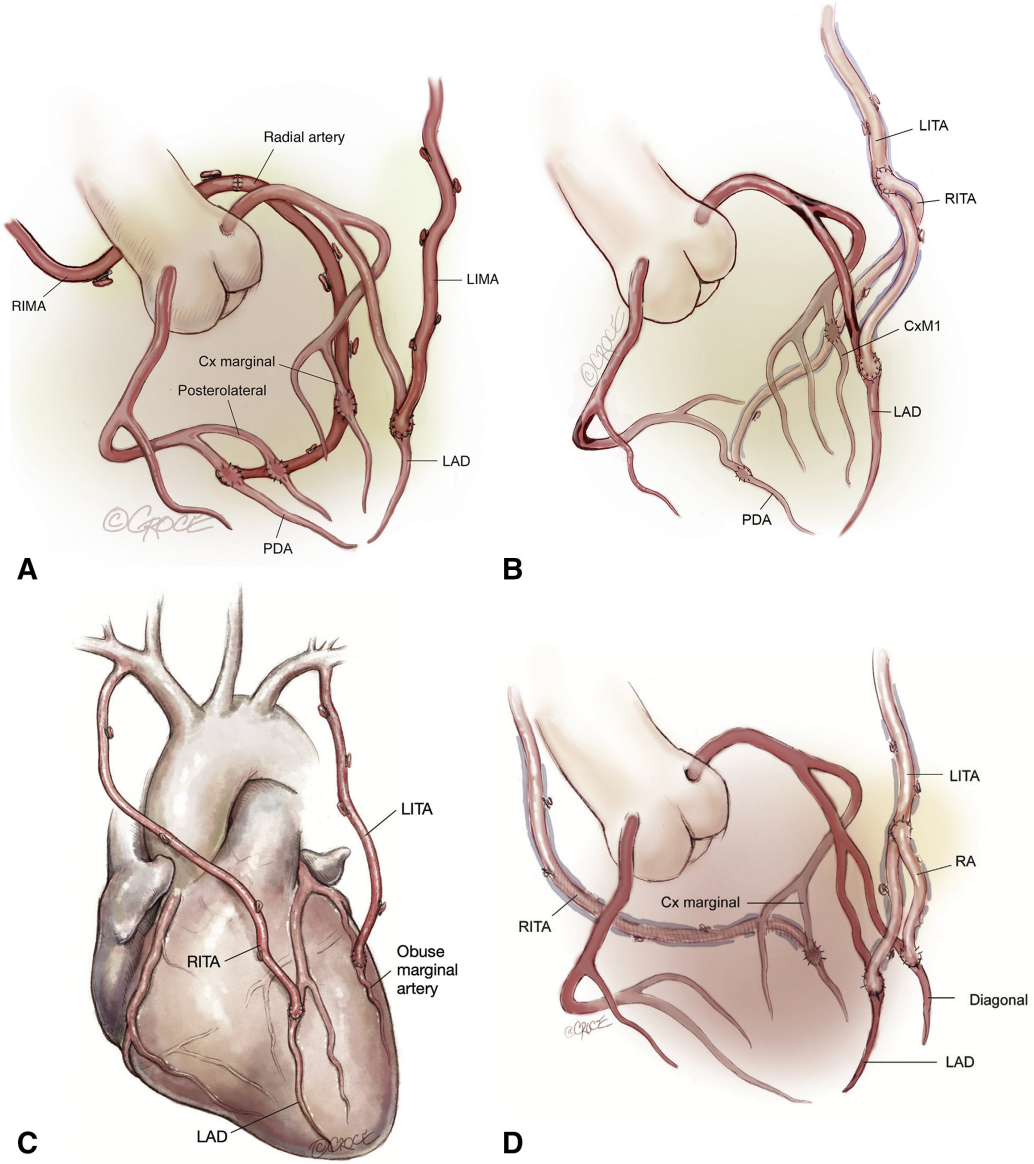
**Surgical strategies to achieve total arterial revascularization**. The RITA or RA can be used to extend the in-situ LITA to reach lateral/inferior or posterior target coronary vessels. (A) *In situ* LITA-LAD and *in situ* RITA-RA extension-lateral/inferior territories. (B) *In situ* LITA-LAD and LITA-RITA “T” graft-lateral/inferior territories. (C) *In situ* RITA-LAD and *in situ* LITA-lateral territory. (D) *In situ* LITA-LAD, LITA-Radial “Y” graft-diagonal vessel, and RITA-lateral territory. RIMA, right internal mammary artery; LIMA, left internal mammary artery; PDA, Posterior Descending Artery; LAD, left anterior descending artery; CxM, circumflex marginal artery; LITA, left internal thoracic artery; RITA, right internal thoracic artery; RA, radial artery. (Reproduced from JTCVS techniques; Dec 2021, Vallely *et al*.; Total-arterial, anaortic, off-pump coronary artery surgery: Why, when, and how [94]).

A further evolution of total arterial vascularization is the no-touch aorta (anaortic) CABG, which implies off-pump CABG with the exclusive use of in-situ grafts BITA, RGEA, “Y” or “K”- grafts with the RA; the RA can also be used to elongate in-situ grafts as shown in (Fig. 1, Ref. [94]). Anaortic CABG likely decreases the risks of intraoperative strokes and renal injury due to atheroembolisms from aortic manipulation. In a meta-analysis of 13 observational studies and 37,720 patients, comparing on-pump CABG, off-pump CABG with a partial aortic clamp (OPCABG-PC), and off-pump CABG without aortic cross clamping (anaortic CABG), anaortic CABG was associated with 78% reduction in the 30-day risk of stroke compared with on pump CABG (OR, 0.22; 95% CI, 0.14–0.33) and 66% compared with OPCABG-PC (OR, 0.34; 95% CI, 0.22–0.52) [95].

## 10. Minimally Invasive CABG and Arterial Grafting

Minimally invasive CABG was described by Calafiore *et al*. [96] when they reported 144 cases of off-pump LITA-LAD bypass using a small (mini) thoracotomy incision; after this report, multiple observational studies have confirmed the safety and feasibility of minimally invasive CABG as an alternative to conventional CABG. McGinn *et al*. [97] reported their experience of 450 patients, where they bypassed LAD with the LITA and used RA or SVG to bypass the lateral or inferior heart territories with the help of apical positioner and epicardial stabilizer through small incisions. They were able to achieve complete revascularization in 95% of the patients, with average number of 2.1 ± 0.7 grafts, 3.8% conversion rate to median sternotomy and a 1.3% perioperative mortality. Tiwari *et al*. [98] presented their experience of 216 cases of minimally invasive direct coronary artery bypass (MIDCAB) through a left anterolateral mini-thoracotomy. They used LITA in all patients and either RITA or RA in a “Y” composite fashion to achieve TAR. TAR was accomplished in 100% of patients with 2.34 ± 0.75 average number of anastomoses, 0% of mortality and deep wound infections at 1-year follow-up, and average hospital length of stay of 4.92 ± 1.46 days with mean intensive care unit (ICU) stay 1.52 ± 0.77 days.

Patient selection and indications for the different types of minimally invasive approaches have been a major challenge for heart teams, as there are no clear guidelines to help with the decision making. The 2018 ECS/EACTS guidelines [79] provided some criteria (Table 5, Ref. [79]) that can be helpful in decision making. Nowadays, MIDCAB grafting can be used in patients with isolated proximal LAD stenosis and also as part of a hybrid approach in selected patients with multivessel coronary artery disease.

**Table 5.** **Considerations for patient selection for minimally invasive artery bypass grafting (Adapted from 2018 ESC/EACTS Guidelines on myocardial revascularization) [79]**.

Considerations for patient selection for minimally invasive artery bypass grafting
CABG through a limited thoracotomy access should be considered in patients with isolated LAD lesion or in the context of hybrid revascularization, if the expertise and institution facilities allow
CABG through a limited thoracotomy access should be considered in the context of hybrid revascularization, if the expertise and institution facilities allow
Hybrid approach, defined as combined or consecutive open surgical and PCI revascularization, can be considered in a subset of patients with multiple coronary arterial disease

CABG, Coronary artery bypass graft; LAD, Left anterior descending artery; PCI, Percutaneous coronary intervention.

## 11. Comment 

Despite the fact that the idea started back in the 1970s, and in the presence of data from observational and randomized studies that support the superior patency of arterial grafts, and even though, it makes more sense to use an artery to bypass an artery, total arterial revascularization is still not widely accepted by the cardiac surgeons especially in the United States. Schwann *et al*. [13] in an analysis of the Society of Thoracic Surgeons database found that between 2004 and 2015 a second arterial conduit was used in 170,677 of 1,334,511 of patients (11.4%; 97,623 RAs and 73,054 BITA; 6.5% and 4.9%, respectively). Those rates are higher in other countries, ≈20%–30% of patients with CABG receive >1 arterial grafts in Europe [99], and in Japan (95.4% of patients received at least 1 arterial graft, and 22.7% received TAR) [100]. This reluctance can be explained by increased technical difficulties, increased operative time, and supposed increased post-operative complications as described by a survey among U.K cardiac surgeons [101]. This might have also affected the training experience of the cardiac surgery trainees and young cardiac surgeons due to lack of enough exposure to MAG procedures during their training as more than 90% of CABG performed in the US and Canada encompasses the use of the LITA and SVG only [11, 12].

In the available literature, surgeons tend to use arterial conduits in patients with younger age, fewer comorbidities like diabetes, ventricular dysfunction, or congestive heart failure, which somewhat can explain the lower use of arterial grafts compared to the venous grafts.

As we presented in this review and based on the available published observational data on the benefit of the multiple arterial conduits use in CABG, we as cardiac surgeon still lack the unbiased and sophisticated data that answers all these questions that were hypothesized from previous observational studies. Only a large, randomized trial will be able to test this hypothesis and provide a solid answer to this important question.

In the next few years, we are anticipating the results of The Randomized Comparison of the Clinical Outcome of Single Versus Multiple Arterial Grafts (ROMA) trail [102], that is designed to compare the use of single vs multiple arterial grafts in an expected sample size of 4300 patients. We anticipate that the ROMA study will add substantial value to the present knowledge.
